# Cocaine-Dosage and Cocaine-Addiction

**Published:** 1887-04

**Authors:** J. B. Mattison

**Affiliations:** Brooklyn, N. Y.


THE CHICAGO

MEDICAL JOURNAL AND EXAMINER.

VOL. LIV. APRIL, 1887. No. 4

### ORIGINAL COMMUNICATIONS.

### COCAINE-DOSAGE AND COCAINE-ADDICTION.

BY J. B. MATTISON, M. D., BROOKLYN, N. Y.

Read before the Kings County Medical Society, February 15th, 1887.

The sad story, in a recent *Record*, of the Russian surgeon's suicide from sorrow or remorse due to his belief that a patient had died from an overdose of cocaine, points a moral, the import of which demands more than a passing notice.

No advent in the therapeutic arena during the last decade has been attended with such varied and extensive claims for favor as cocaine. Its marvelous effect in ophthalmic surgery aroused a spirit of experimental research in other directions which has added largely to its well-proven power for good; but, as has been well observed, a potency for good implies a potency for harm, and the risk impends of its ardent advocates being carried by over-enthusiasm beyond the limit of a safe regard for the welfare of their patients or themselves, that may imperil an otherwise well-founded success.

Surely it is, in the writer's opinion, full time to draw the line; to re-voice a warning as to the use and abuse of this valued, but, at times, toxic drug, lest the roll of alarming, dangerous and fatal effects from its ignorant or incautious use be sadly extended, and a reaction ensue that, by creating distrust within and without the profession, will damage its good repute and hinder its use in cases where it would be almost certain of serving us well. And the need of this seems all the more called for in view of opinions expressed, during the past year, in certain quarters, affirming the harmless character of cocaine —opinions which, I am convinced, are at variance with well accredited facts, and should not be allowed to pass uncontradicted.

Cocaine seems to have secured for itself a more than usual share of attention aside from the professional press. One metropolitan daily, in particular has again and, again given its columns to a discussion of the topic, and in a somewhat lengthy article not long ago, an "eminent but unnamed specialist"—Dr. Francke H. Bosworth—was reported as saying "there is not a well-authenticated case on record, as yet, where cocaine has effected injury."

In view of cases cited in this paper, and others elsewhere recorded, such a statement is no longer tenable, and any conclusion based thereon as to the harmless nature of cocaine is misleading and incorrect.

And the evidence herewith presented weighs even more heavily against an assertion by Dr. Wm. A. Hammond, at a recent meeting of the New York Neurological Society, in the course of his "Remarks on Cocaine and the so-called Cocaine Habit," when, after telling his taking of eighteen grains at a subcutaneous dose, he asserted "he did not believe any dose that could be taken was dangerous!" What might be the outcome of such an opinion put in practice? The Russian surgeon's error of judgment, fatal to his patient and himself, was largely due to his reliance on the asserted use by other surgeons of large doses without ill-effect. Might not a like result follow an incautious dependence on Dr. Hammond's disbelief in the toxic power of cocaine? The *Record* well said of Prof. Kolomnin's case: "The experience, though so sad, may not be without its lesson"; and puts a very pertinent query as to whether "there are not other surgeons who could report very serious, if not fatal, results from injudiciously or ignorantly using too large a dose of cocaine.

Fifty cases herewith noted attest a power in this drug on some patients that warrants caution with all.

"A young woman, aged twenty-three, was sent to Prof. Kolomnin, and found to have a large ulcer of the rectum, which was diagnosticated to be of tuberculous nature. He decided to scrape and cauterize the lesion and to use cocaine anæsthesia during the operation.

"In order to produce anæsthesia, he had fifty grammes of a five per cent. solution of hydrochlorate of cocaine prepared; of this, thirty grammes were brought into use, containing exactly twenty-four Russian grains of the salt, or twenty-three English grains—the Russian grain is exactly one-sixteenth of a gramme—six grains being injected at a time into the rectum. After the third of these injections, it was found on examination that the part was still sensitive. A speculum was then introduced, the ulcer dabbed with a dry sponge, and then the fourth injection given, making twenty-four grains in all. After this the parts were tolerably anæsthetic. The ulcer was scraped, and a tampon saturated with oil inserted. The pulse was then accelerated. During the operation the patient groaned, so that even the twenty-four grains had not produced complete anæsthesia.

"After the operation, Kolomnin went round his ward, and in three-quarters of an hour a message was sent to him that the patient was very low. He found the pulse very weak, the face and hands cyanotic, and the respiration labored. He considered that she was in a toxic state, and used every means to bring her round, Prof. Sushchiniski being also invited to a consultation. Faradization, artificial respiration, hypodermic injection of ether, administration of ammonia, tracheootmy for the inhalation of oxygen, stimulating and nutrient enemata—all were tried, but without success. Kolomnin had no doubt that death was due to cocaine."

Dr. W. H. Long, U. S. Marine Hospital Service, reports in the *American Lancet* the case of a man aged thirty-three, to whose larynx he applied, three times, a four per cent. solution of cocaine. Prompt relief was given, but three and one-half hours later the patient was found unconscious; breathing, labored; respirations, twenty; pulse, ninety; general condition, one of profound anæsthesia. Diagnosis: cocaine poisoning. Several doses of whiskey were given subcutaneously. In half an hour, consciousness was partially restored, then gradual and full improvement, save a feeling of great exhaustion.

Four days later cocaine was again used. Thinking the former toxic effect due to swallowing some of the solution, and probably absorption by larynx, extra precaution was taken to have it expelled and the pharynx well rinsed. Two applications of a two per cent. solution were made. Relief was again complete, but three and one-half hours after the patient was in the same condition as before, except that the anæthesia was not so profound. Frequent injections of whiskey were again used with partial success—he could swallow and answer questions—but, soon after, he suddenly ceased to breathe. The heart beat a short time longer. All efforts at resuscitation failed. The probable immediate cause of death was paralysis of the respiratory center due to cocaine.

Dr. F. M. Thomas, Leonardsville, Kansas, reported to Prof. R. Ogden Doremus, as follows:

"Friday morning, October 23d, 1885, I was called to see Mrs. ——, aged thirty-nine, whom the messenger reported as dying. I found her unconscious; breathing heavily and irregularly, pulse thirty-five, intermittent; temperature normal; left pupil largely dilated, right natural; right arm and lower limbs motionless; face spasmodically drawn upwards toward the dilated eye.

"Spasmodic action of the left arm and upper part of the body came on regularly at intervals of a few minutes, during which she clutched the bed-clothing, and seemed to be trying to vomit. Twice during my attendance she ejected small portions of the previous evening's meal. Salivation was excessive; she retained a dorsal decubitus; would not lie on either side. Heart seemed almost exhausted.

"I saw her at 5 A.M., and was with her nearly all the time till she expired, apparently completely exhausted, about 8:30 A.M."

On inquiry, the Doctor learned that Mrs. —— had been freely using a four per cent. solution of cocaine, for toothache, due to several much-decayed left upper molars. His diagnosis was cocaine poisoning.

Dr. Knabe, of Berlin, records the case of a girl aged eleven, who was given four to twelve drops—the exact amount was not determined—of a four per cent. solution of cocaine, by injection over the deltoid, to remedy frequent fainting fits—she having cardiac degeneration, *a sequela* scarlatina. In less than forty seconds the girl took a deep breath, became deadly pale, and dropped unconscious. One minute later she was dead.

In the Australasian *Medical Gazette*, August, 1886, Dr. W. E. Ramsden Wood reports this case. "A. B. suffered from neuralgia, due to a defective tooth. Extraction being impracticable, cocaine—amount not stated—of a ten per cent. solution was injected, with prompt relief, lasting some hours. Next day, the pain being very severe, the patient sent to his chemist for a similar solution, and three minims were injected, but without the desired effect; he returned it to the chemist and asked him to make it stronger, which he did, making it twenty per cent. He brought this to me, but omitted to tell me that the solution was double the strength of that which I had used. He told me that three minims had not given him the relief that he had experienced from mine; I therefore gave him four minims of what I belived to be a ten per cent. solution, and within five minutes he became restless and inclined to vomit; he then began to feel a sensation of pins and needles in the left hand and arm, which rapidly extended to the right side. This was speedily followed by contraction and rigidity of the fingers, arms and legs; there was also a tendency to opisthotonos. His pulse became extremely rapid and feeble, his face livid, and the muscles of his mouth and cheeks strongly contracted. His respirations were short and convulsive, his feet and hands very shortly became cold, and a profuse perspiration broke out on his head and face.

I first gave him half a tumbler of brandy, followed at short intervals by drachm doses of spiritus ammon. aromatic, and applied strong mustard over the cardiac region, and used friction to the upper and lower extremities; at the same time I let him inhale a few drops of chloroform to try and check the spasmodic contractions. After continuing these remedies for nearly an hour, the pulse began to improve, the color to return to the face, and the rigidity of the muscles lessened, but returned immediately after I stopped the friction. At the end of two hours he improved more rapidly, but felt somewhat drowsy, and it was not until about four or five hours that all the symptoms had subsided.

C. S. Kilham, L.R.C.P., Sheffield, England, read before the Sheffield Medico-Chirurgical Society, November 25th, 1886, this history of a case: On November 9th, 1886, at twelve noon, John B—— accidentally took four and four-fifths grains of cocaine hydrochlorate in the form of solution. At 12:30 he was seized with severe cramps in the stomach, nausea, throbbing and feeling of bursting in his head, failure of eyesight, loss of use of his legs, incoherence of speech and confusion of ideas, and drowsiness, but could always answer questions when aroused. No delirium; appeared as if drunk, and got quite helpless. Brandy was given to him and he vomited after it, but only the remains of food. About 12:50 he commenced sweating most profusely, shirt, etc., being soaked through, perspiration streaming down his face and body, and his head steaming. Pupils were normal and equal. No loss of taste. The sweating lasted some time and was succeeded by very severe prostration, shivering and feeling of impending death. At intervals the patient had severe cramps in the stomach, with retching and vomiting of a quantity of clear mucus, which relieved the pain. About 1:15 P. M. the pulse became intermittent—missing every fifth beat. This was accompanied by cyanosis of the face and intense feeling of suffocation over the cardiac region. Relief was afforded by sinapisms. The pulse varied from eighty to eighty-six, never more, and became gradually regular. About 1:45 P. M. he began to have cramps in the legs and feet—especially on dorsal surface of right foot—and tingling and numbness in both hands. Later on the pupils became dilated. The vomiting and cramps ceased about 4 P. M. —unless food were taken—but the drowsiness, throbbing of head and prostration continued up to 6 P. M., when the patient began to get warm and feel relieved. The improvement continued, and he could be moved at 8:30 P. M. There was great weakness, with swimming of head all night.

Next day there was still weakness, continual vomiting, a dry, leathery feeling in the mouth, with loss of taste, partial loss of power in the legs, and tingling and numbness of the fingers, especially of the right hand. These symptoms commenced nearly thirty-six hours after taking the cocaine, and most of them disappeared in twenty-four hours. The loss of power in the legs lasted three days, and the tingling and numbness of fingers longer. He was not able to write a letter until the sixth day, as he could not feel the pen between his fingers before.

An emetic was at first given, with sinapisms over the heart and stomach; afterwards, warmth and stimulants—principally compound spirit of ammonia.

The patient was in the habit of taking one-fourth grain of cocaine for neuralgia of the stomach. The most remarkable symptoms were the severe sweating, the intense prostration and the intermittent pulse.

Dr. Geo. Elder, Nottingham, Eng., in the *Lancet*, October 30th, 1886, says: "Preliminary to opening a superficial abscess, twelve minims of a freshly prepared ten per cent. solution were injected under the skin; three or four minutes after, syncope supervened, followed by twitchings of the face, falling of the jaw, coldness of the body, clammy perspiration, lividity of the face—in fact, all the appearances of imminent death. The patient was several minutes in recovering consciousness, and during the remainder of the day felt very prostrate." Dr. E. Adds: "Several similar occurrences have been noted, showing that cocaine is not so innocuous as has been generally supposed."

James Leslie Callaghan, in the London *Lancet*, June 12th, 1886, reports as follows: "A patient of mine who was suffering from toothache resulting from a hollow tooth, applied some of a four per cent. solution of hydrochlorate of cocaine to the tooth and gums. He did not spit it out, but, according to his story, swallowed from twenty to thirty drops. Within half an hour he was seized with a feeling of faintness and giddiness, then an attack of palpitation of the heart came on, and he complained of tingling and numbness, dryness of the throat, and a sensation of heat and flushings moving over the body, but especially on the spine. Suddenly a rash like scarlatina made its appearance over the body; vision was somewhat dimmed. I immediately gave him a strong dose of mustard and warm water, which did not cause emesis. I then administered twenty grains of sulphate of zinc, but without effect; it was only by repeating the dose that vomiting took place. The patient was relieved for a few minutes and seemed brighter, but the symptoms soon returned, and he felt so weak that he thought he was dying. I held some strong ammonia to his nostrils, but he said he could not smell it. I kept walking him about, but his legs tottered so much I had to support him. He constantly felt a desire to have the use of his bowels and bladder. The pulse became fast, weak and intermittent; the mind remained clear."

Dr. Fred'k S. Williams, of Puyallup, Washington Territory, reports in the *Medical Record*, September 25th, 1886, this case: A lady on whom he wished to operate for a lacerated cervix, had a pledget of cotton saturated with a twenty per cent. solution of cocaine placed over the cervix, and four minims of the same solution injected on each side of the wound. "In about a minute and a half the patient began to speak as with an effort, saying 'I feel so faint,' and gasped as if struggling for breath. She was immediately placed on her back, with head lowered, and told to breathe deeply. She obeyed for a few times, then recommenced her gasping, which she continued for about a minute. Then followed shallow breathing for four or five minutes, when she began to rally a little, and the breathing became gradually stronger but irregular.

"Her pulse at first was very rapid, irregular and weak, then became, during most of the time of the shallow breathing, almost imperceptible, gradually returning with the approaching normal respiration.

"Consciousness at once was dulled, and during the period of the shallow respirations was completely lost.

"At the end of about ten minutes she rallied, pulse, respiration and consciousness becoming normal."

Myerhausen relates the case of a "girl, twelve years old, in whom two drops of a two per cent. solution were instilled in the conjunctiva four times, at intervals of five to eight minutes. In all, only a little over one-tenth of a grain was administered, of which certainly one-half must have been lost through the tears. Immediately after the operation, the child commenced to complain of headache, which became more and more severe until it was almost unbearable. Nausea and vomiting persisted through the entire day. The patient was greatly prostrated; stumbled in walking; speech was almost entirely destroyed, as though the tongue were paralyzed. These symptoms of poisoning lasted all through the night, in which no rest was possible, and gradually disappeared towards the evening of the following day."

Dr. Robert Newman, New York city, kindly sent me this report: Patient, a female, aged thirty-seven, was treated for chronic cystitis, by washing out and dilating the bladder daily. To allay the pain, fifteen minims of a four per cent. solution of cocaine were injected per urethra. This, increasing the drug a little each day, was used three times. After the third injection, while the cystic pain was allayed, a severe headache ensued, which persisted for several hours. On the fourth day, having increased the cocaine to twenty-five minims, "while still washing out the bladder with *hot* water, a piercing pain in left temple occurred, running round the back of the head in a circle, and feeling as if the top of the head would split open. Pupils dilated; expression anxious; restlessness marked. More than a week passed before all toxic traces ended. There can be no doubt cocaine caused the trouble, and the symptoms were alarming."

In the London *Lancet*, 1886, is recorded the case of a female, aged twenty-five, who had a watery solution containing fifteen centigrammes of hydrochlorate of cocaine injected into the nose. In twenty minutes giddiness, weakness and impaired vision ensued. A little later she was semi-comatose, with slight dyspnœa and pulse uncountable. These symptoms disappeared in three hours under friction and internal stimulation.

Dr. Schilling in the *Pharmaceutical Journal*, records a case in which the injection of six drops of a two per cent. solution into the gums of a woman, aged twenty-six, to prevent pain of extracting a molar, was followed by toxic symptoms, of which facial rigidity, deafness, blindness, complete loss of motion and sensation, and unconsciousness for a half-hour, were the chief. They subsided after inhaling nitrite of amyl.

Dr. Heyman reports a case in which the effects following the use of cocaine closely resemble that noted by Myerhausen. The patient, a boy, had a solution of cocaine liberally applied to his pharynx and larynx. Toxic symptoms soon set in. He was apathetic with speech and walking disturbed. Pulse and respiration increased. Temperature rose to 100¾. Five hours after, patient could not walk. Symptoms persisted ten hours.

Dr. Schwarzbach, Australasian *Medical Gazette*, January, 1886, reports the case of a lady who used cocaine, locally, for pain in the eye. She suddenly became very ill; stupor, pallor, slow pulse and cold perspiration. Under wine and strong coffee, recovered in a few hours.

G. Bockl observed alarming effects follow an injection of six drops of a two per cent. solution into the gums. In ten minutes patient became unconscious, with gaze fixed, vision defective and delirium. Nitrite of amyl gave relief.

Dr. Landesburg, New York city, used two grains subcutaneously, as an experiment, on himself. In less than two minutes he felt his heart beating violently and blood rushing to his head, quickly followed by fullness and roaring in the latter, and noises in the ears. Thought was confused, volition impaired. Great restlessness, and numbness with twitchings were felt in toes and fingers. Nausea and epigastric pressure marked. Face very pale and covered with cold sweat. Pulse feeble, eyes sunken, pupils dilated, vision dim. In thirty minutes, took to bed with nausea, headache and general prostration. Recovery followed a night's sound sleep.

Drs. Bardet and Meyer, assistants of Dujardin-Beaumetz, anæsthetizing, for experiment, their own skin, observed, half an hour after the injections, dilated pupils and comatose symptoms. One of them fell in a state of vertigo, with pallid face and extreme heart weakness. These toxic symptoms followed hypodermic doses, never exceeding one-third of a grain.

Dr. Ziem, of Dantzic, in 1885, reported a case in which a solution applied to the eye caused pallor and embarrassed breathing, and said that, up to that time, seventeen cases had been cited in ophthalmological literature, in which toxic effects followed the use of cocaine. In three, by injection; fourteen applied to the eye. Pallor, giddiness, dyspnœa, malaise, apathy, great prostration, tottering gait, difficulty of speech, mental confusion and extraordinary restlessness were symptoms noted in both strong and feeble men and women.

Dr. G. W. Kennicott, in the Chicago MEDICAL JOURNAL AND EXAMINER, October 20th, 1885, reports: A young woman, aged twenty-five, of good constitution, had been using, per medical advice, a two per cent. solution of cocaine for hay-fever. The supply becoming exhausted, she procured two 5 grain vials of the muriate, full strength, and applied two-thirds of the contents of one bottle to both nostrils with a small glass insufflator. In twenty minutes she became dizzy, vision dark' and a sinking sensation occurred, with great weakness. In halt an hour she was semi-comatose, pulse scarcely countable, so rapid and weak; pupils widely dilated; speech and swallowing difficut; dyspnœa; nausea; throat dry; teeth chattered, and she shivered with cold. Later, drowsy; eyes closed; face muscles affected; weakness extreme, she could not support her head. She recovered in three hours under brandy, ammonia, digitalis, heat to epigastrium, and heat and friction to extremities.

Dr. Geo. J. Engelman, in the *Medical Review*, June 13th 1885, records these cases: Mrs. C., aged twenty-eight, in fair health, at 5 P. M. took one-sixth of a grain by the mouth; one hour later this dose was repeated, and soon after she felt a tingling in her fingers, hands and wrists, with discomfort and oppression about the chest, and vomiting the moment she turned in bed. At 7:30 she took a third dose, same amount, and in fifteen minutes was excessively restless, great difficulty of breathing, tight band-like feeling about chest, faint and felt as if dying. At 8 o'clock still faint, was dyspnæic, and tingling had extended to feet and legs. At 8:15 tingling gave way to numbness, beginning in hands and extending to feet; "became perfectly still, as if breathing her last;" quite numb and stiff; thumbs adducted; pulse feeble, frequent, irregular and intermittent.

These toxic symptoms subsided after one-sixth of a grain of morphia, hypodermically.

Mrs. F., aged thirty-five, enciente, took forty drops of a four per cent. solution to relieve nausea. Immediately she felt a complete numbness along the tongue and throat; to test the feeling she bit her tongue, and found it perfectly numb. She became weak, perfectly relaxed, with oppression about the heart, and felt as if dying. In twenty minutes the entire body became cold and numb. Pulse feeble and very rapid. Heart felt as if constricted by an iron band, and "hammered loudly at a fearful rate." Symptoms persisted several hours.

Dr. Litten, at a meeting of the Berlin Medical Society, November 4th, 1885, in a debate on the action of this drug, cautions against its too general use. He said that among other ill-effects known to occur after an injection are attacks of mania, sometimes very violent, which may prove dangerous; and he asserted the various toxic effects, in some individuals, reach such a high degree that actual danger to life seems to threaten the patient. The three cases next cited are of interest in this regard.

Dr. Geo. T. Stevens, *Medical Record*, January 17th, 1885, reports that he injected four minims of three and one-half per cent. solution under the conjunctiva of a strong man. In eighteen minutes "violent convulsions set in, attended with desperate struggles to breathe. The face became livid, consciousness was lost, and the patient became uncontrollable. After struggling in an easy-chair for some time, he arose in a state of frenzy and struck violently about. Stimulants were administered, and the most alarming stage of the paroxysm ceased after a duration of nearly twenty minutes. Fully half an hour, however, passed before we could regard our patient as beyond danger. I believe that this paroxysm was the manifestation of the toxic influence of the drug."

Dr. Robert Newman, of New York, has reported to me the case of a gentleman, aged forty, in whose urethra a physician injected one drachm of a cocaine solution—strength not stated—prior to cutting the meatus. In half a minute, patient's face flushed, he felt a general pricking sensation, followed by a piercing sting in his temple, violent headache and great excitement. Then he became maniacal, and under the delusion that he had been attacked by a robber, sprang from his seat, seized the doctor by the throat and began to beat him. The delirious excitement persisted three hours.

A well-known physician of this city gave me his experience with cocaine. Suffering from an attack of otitis media, he used freely, by advice of his medical attendant, a ten per cent. solution in the ear. It caused flushed face, quickened pulse, and breathing—the former, 130—wild look, fixed gaze, hallucinations and delusions—the latter homicidal—attempting assault on a near relative—which persisted three hours, followed by decided depression.

Dr. J. P. Knoche, in the Kansas City *Medical Record*, December, 1885, reports the case of a man, aged twenty-three, to whom he gave cocaine, hypodermically, for anæthesia, using, in several injections, within thirty-five minutes, about two and two-fifth grains. In seven minutes the patient was cold, and sensation lost in hands, forearms, chest and legs. In twenty minutes breathing was difficult, interrupted, sighing. Pulse almost imperceptible, intermittent and very rapid ; lips and skin generally pale and cold. Patient was semicomatose for a time. Numbness in extremities lasted four hours ; imperfect palmar sensation ten hours. Nine hours after, severe renal pain and copious diuresis ; the tremor and weakness continued twenty-four hours. Symptoms gradually decreased under free alcoholic stimulation.

Dr. H. J. Boldt, New York city, reports four cases of toxic symptoms from cocaine injections. He injected fifteen drops of a four per cent. solution to relieve an attack of supra-orbital neuralgia. Immediately the patient's face became red, there were dizziness and dyspnœa, his pulse became frequent and feeble, and feeling of oppression about heart occurred. In three minutes the patient fell unconscious and breathing ceased, so that, for fifteen minutes, artificial respiration was required. Then respiration returned, but only five or six per minute, deep and sighing. Heart sounds and pulse very weak and frequent ; pupils widely dilated ; gaze fixed. Shortly before and directly after return of consciousness, there were convulsive twitchings of the upper extremities. Ten minutes later, speech was confused and incoherent. In an hour the patient was able to walk, but his gait was unsteady. Neuralgic pain did not return for three days. At the patient's request, ten drops of the same solution were again injected, and like symptoms resulted, but they were not so prolonged. Five or six drops of this solution, in this patient, caused flushing, dizziness and dyspnœa, with constricted feeling about chest, continuing half an hour.

II. Female, aged thirty-three. Eight drops of a four per cent. solution were injected for local anæsthesia prior to operation for lacerated cervix. It caused flushing, followed by dizziness, dyspnœa, nausea, deathly pallor and cold sweat. Pulse frequent and feeble; pupils dilated. Symptoms subsided in half an hour, leaving patient so weak that operation was deferred.

III. Female, aged thirty-three. Neuralgic headache. Eight drops of four per cent. solution were injected over seat of greatest pain, causing dizziness, dyspnœa, quick, weak pulse, distress in chest, especially about the heart, deathly pallor, nausea and sweating, persisting more or less for forty-five minutes.

IV. Female, aged twenty-six. Five drops of a five per cent. solution caused, three minutes after injection, giddiness, palpitation, quick pulse, languor, and temperature rose to 100 8-10 in fifteen minutes.

Dr. F. De Havilland Hall, London, reported the case of a lady, aged fifty-six, to whose nostrils he applied a ten per cent. solution, by spraying. In a few minutes patient complained of cramp-feeling in throat; became very excited; face ashy hue; hands cold; pulse very frequent, and distress was so great that chloroform was given, which relieved the spasm, but she was not able to leave until after four hours.

Knapp noted headache, vertigo, nausea, tottering gait, skin pallor and cold sweat from hypodermic injection of thirty-five drops of a four per cent. solution, with instillation of a few drops of the same in the conjunctival sac.

Reich reported two cases, both females, aged ten and sixty, in which toxic symptoms followed the use of fifteen drops of a two per cent. solution.

Bellyarminoff, of St. Petersburg, observed five cases in which a four per cent. solution to the eye caused headache, vertigo, nausea and vomiting.

Alex. Thompson, M.B., Huntly, Eng., reported this case: He applied a few drops of a fresh two per cent. solution to the conjunctiva of W. R., aged twenty-five, perfectly healthy, prior to removing a fragment of steel. In two minutes, patient became deadly pale, reeled, and would have fallen if not supported. Was quite pulseless, and with difficulty was brought round. Fully half an hour passed before he could go home, and even then was giddy.

Dr. Grosholz, Towyn, Eng., observed a healthy farmer to whose eye three drops of a four per cent. solution were applied, causing pallor, profuse sweating about head and neck, irregular pulse, embarrassed respiration and impending syncope. A stimulant was given, but it was several minutes before the pulse became regular and consciousness was regained.

Dr. Edward Bradley, New York city, noted this case: A professional gentleman, in perfect health, had a four per cent. solution freely used in the filling of a carious tooth. Toxic symptoms soon appeared, the most noted being facial paralysis on the right side. "This condition undoubtedly began its development much earlier than the time of its discovery, as it went on its course of extension for the two succeeding days, involving every function on the right side of the head, rendering me deaf, and unable to close the eye. The brain was depressed so as to destroy all continuity of thought, and I was unable to read or exercise any mental function whatever." The paralysis remained stationary ten days, then slowly lessened, but had not entirely gone at end of six weeks.

Smidt, Ranc, Obersteiner and Blumenthal have noted, after an injection of cocaine, dizziness, agrypnia, muscular twitchings, increased reflex excitability, hallucinations and mania.

Dr. Chas. H. Hughes, St. Louis, editor of *Alienist and Neurologist*, wrote me: "I know of a case where one grain of cocaine paralyzed the heart so effectually that the pulse became imperceptible for a few seconds, and only my presence with my battery, which was in the room, and ammonia, and a morphia and strychina hypodermic, saved the patient."

Germane to the subject of acute cocaine toxæmia is that of cocaine-addiction—these notes are preliminary to a more extensive paper on cocaine inebriety—the existence of which Dr. Hammond denies. He took a half dozen doses, at intervals of one to four days, and says "he acquired no habit." But to argue from that, no danger of addiction, is absurd. Such evidence is worthless. Dr. Hammond might do the same thing with morphia—more, he might take morphia, subcutaneously, daily, for a month or two, without creating a "habit"—albeit its ensnaring power is well admitted—and yet that would not prove its freedom from danger. Not at all; it would merely show his exceptional strength to resist—many, under a like pressure, would surely succumb.

Supporting this opinion, I quote from the last report of Dr. Orpheus Everts—Cincinnati Sanitarium—a gentleman well-known in alienistic circles, which report was kindly sent me after my paper was written—who says: "A distinguished physician of New York has recently reported personal experiences tending to discredit the claim that a cocaine habit, corresponding to the morphine habit, is acquirable. The judgment of this distinguished physician is based upon the evidence of personal experience reported by himself, he having failed to acquire the habit, or any especial fondness for the specific effects of the drug, experienced by the hypodermic injection of one, two, three, and finally eighteen grains of the salt, on five or six different occasions in the evening before going to bed.

"But for the great reputation of this physician as an author and observer of facts, this denial would have but little weight. The testimony is both bad and insufficient. Bad, because reported by himself—the testimony of an intoxicated person respecting his experiences while intoxicated being proverbially untrustworthy—and insufficient, because the experiment was not continued long enough. Many instances might be cited of total failure to establish the morphine habit or habitual drunkenness, by the use of six or seven doses of morphine, or six or seven drinks of whiskey, one a day for six or seven days in succession. It is often the case that such experiences end with disgust for the drugs used, instead of desire to continue their use. There is also much and accumulating testimony, by competent observers, to the fact of such a habit as is alleged, respecting cocaine, which a single opinion will not invalidate, however worthy of consideration."

Cocainism is not the outcome of using the drug at long intervals. Its transient effect and the demand of an impaired nerve status compel frequent taking—more than alcohol or opium—so that habitues have been known to take it ten, twenty or more times daily, and it is this—growing by what it feeds on—that tends to create and continue the disease.

In the early days of chloral, one point claimed in its favor was a freedom from risk of "habit," a claim long ago exploded, as cases of chloralism well prove, and yet, I venture to assert, there are more cases of cocaine taking in this country to-day, less than three years since its arrival, than of chloral after a period more than six times as long.

Dr. Hammond says there may be instances of cocainism as rare as chronic tea-taking, and of cases with or after habitual alcohol or opium-using, but, as for quitting the drug, he believes every cocaine-taker could if he would.

The same opinion regarding opium obtains among some medical men, and the only effective argument against such a fallacy is to place those who hold it under power of that drug, and then have them prove their precept by their practice.

While admitting that most instances of cocaine-taking are, for obvious reason, in those who have been, or are, alcohol or opium habitues, especially the latter, I maintain there are cases of pure primary addiction, and that the number is increasing, at home and abroad. Foreign writers have noted them, and they will figure in our records. Notes of one such are here given; others are at command.

I am indebted to the courtesy of Dr. J. E. Clark, Professor of Chemistry in the Detroit College of Medicine, as follows: "In July, 1885, a young man applied to me for relief for what he called a 'severe and persistent case of hay-fever.' He had consulted a large number of physicians without obtaining relief from the irritating and annoying acrid discharge from the nostrils. He claimed he only wished relief for a time until he could make arrangements to leave for the West, as he had lost hope for anything like a permanent cure in this climate.

I prescribed the following:

| | |
|---|---|
| Cocaine Muriat | Eight grains, |
| Aquæ Camphoræ | Six drops, |
| Aquæ Destill | One ounce. |

with directions to pour a small quantity in the hand, and snuft occasionally.

Three or four days later he appeared at my office, his face radiant with hope that he had found a specific, and remarked that "he had felt better the last few days than for a year." He had exhausted his medicine, and I consented to his having the prescription refilled. I heard no more of the case, until one day in November I was in a drug store, and was asked by the proprietor 'do you know Mr.—— is using about eight dollars worth of cocaine a week?' I expressed my astonishment, forbade any further sale, and at once called but failed to find the young man. I then drove to his parents' residence and asked them if they were aware of their son's excess in this matter. They did not know of his addiction to the cocaine, but had noticed for some time peculiar symptoms which alarmed them. I explained the circumstances fully and added my belief that no serious results would ensue provided total abstinence should follow. The sequel shows, however, the 'habit' had gone too far to be easily eradicated, as the young man went on until he became temporarily insane, had to retire from his business, and be under strict supervision until the craving disappeared, a space of about three months." The doctor adds—"this case has furnished me such *positive proof* of a lurking danger in the use of cocaine, that all the negative evidence or hypothetical assumptions published or adduced cannot have the least impression in causing me to relax for an instant the most rigid supervision of every patient to whom I in any manner administer or apply the drug."

The following report, kindly sent me by Dr. Douglas Schoolfield, Newport, Ky., well shows the ill-effect of cocaine on patients addicted to morphia.

Mr.—— had been an opium-habitue for several years, from morphia given subcutaneously to relieve sciatica.

In May, 1885, taking ten to fifteen grains daily, with nervous and digestive systems impaired, but weight nearly normal and mind clear and vigorous, he came under the doctor's care. Cocaine being then in rising repute as a cure for morphia taking, he was given half a grain subcutaneously, knowing, unfortunately, what it was. Nausea, pallor, rigors and cold sweat soon ensued, followed by flushed face, brightened eyes and a feeling "never so good in all his life." He was delighted, talked incessantly, and declared himself, mentally, the peer of Spurgeon; physically, equal to Samson. The stimulant effect continued two hours, followed by lassitude and sleep. The cocaine was given at irregular, increasing intervals for two weeks, when an effort was made to quit. Two days later, he left for a summer resort and was lost sight of. The next heard of him was through the town druggist, who remarked that he thought Mr.—— must be "getting a 'corner' on cocaine," as he had ordered his entire supply and the address of his wholesale house. He was written to and urged to place himself under proper medical care, but no more was heard of him for two months, when the Doctor was sent for, told he had been brought back, and given this history. On starting for his summer trip he procured a supply of cocaine and began taking it himself, several times a day. In a few days he talked and acted strangely, slept little, appetite failed, and he grew worse daily. An effort was made to withhold it, or substitute morphia, but he resisted both and raved like a madman. He had always been kind and even tempered, but now became irritable and abusive; had hallucinations and homicidal delusions; would leap from bed, rush to window, raise sash and gesticulate wildly at a fancied foe. Calmed, he would be quiet a time, and then break out in the loudest abuse of some friend present, declaring him in league with the devil for his harm.

The doctor was warned as to entering his room, and, proceeding with care, the patient was found in fighting form, with a long-necked bottle ready for battle. Addressed kindly, his suspicions were disarmed, he abandoned his hostile attitude, apologized, and declared himself quite mistaken. "His condition was pitiful indeed. Constant vigil and loss of sleep had made him a wreck. He was pale, thin, and haggard; ate nothing and slept none; was a prey to distorted fancy, a victim of unrest."

Under proper treatment, he partially recovered, and was placed in sanitarium care. Six weeks after he was discharged, but in bad mental condition, morose and melancholic. He soon became violent and threatening, and was again taken to an asylum, where he now is, improved and improving.

My experience with a number of cocaine cases makes to me two things certain: There is a pernicious power *per se* in this drug, and it finds in the opium-habitue a peculiar condition that specially favors its ill effects, making it, for such patients, as has well been said, the "Devil's own device" to still further enslave.

And this opinion is that of others, for it is the testimony, without exception, so far as I know, of those who have had to do with this disease, that as an intoxicant cocaine is more dangerous than alcohol or opium, and that inebriety resulting from its use is more marked and unyielding than any other form.

Dr. Shrady—editorial, *Medical Record*, Nov. 28, 1885—says: "To some persons nothing is more fascinating than indulgence in cocaine. It relieves the sense of exhaustion, dispels mental depression, and produces a delicious sense of exhilaration and well-being. The after-effects are at first slight, almost imperceptible, but continual indulgence finally creates a craving which must be satisfied; the individual then becomes nervous, tremulous, sleepless, without appetite, and he is at last reduced to a condition of pitiable neurasthenia."

Dr. Alex. B. Shaw, physician to St. Vincent Asylum for the Insane, St. Louis, asserts: "Once a man flies to cocaine for relief from 'cares that annoy,' he generally continues with such rapid strides towards such complete subjugation to its bewitching thraldom as but few will ever be rescued from by any power of will which they be able to bring to their aid."

Dr. Everts writes: "It is not only not an antidote to opium-poisoning—or, more properly speaking, the organic demand for such drug-effects as have been acquired by use—but is itself a fascinating and dangerous intoxicant, the effects of which may be more difficult to counteract and renounce than are those of opium or its derivatives."

Dr. Hughes declares it "a remedy to be used with extreme caution and prudence internally, and the large doses reported as having been given are not ordinarily safe. It will bear watching. It crazes and kills quicker than opium. The possibilities for immediate harm are not only great, but the likelihood of remote damage, when tolerance is established, is not small. The cocaine-habit, more pernicious than the morphine-neurosis, is the certain entailment of its frequent administration, and its thraldom is far more tyrannical than the slavery of opium."

Erlenmeyer calls cocaine the third scourge of humanity—alcohol and opium being the first and second—and Erlenmeyer is right, as to toxic neuroses. He says: "Its characteristic effects are vaso-motor paralysis, accelerated pulse, profuse sweats, dyspnœa and syncope, failure of general nutrition, eyes sunken, skin cadaveric, with mental trouble that sometimes needs restraint," and I am positive, from cases under my care, that he is correct.

I think it for many—notably the large and enlarging number of opium and alcohol-habitues—the most fascinating and seductive, dangerous and destructive drug extant; and while admitting its great value in various disordered conditions, earnestly warn all against its careless giving in these cases, and especially insist on the great danger of self-injection, a course almost certain to entail added ill.

To the man who has gone down under opium, and who thinks of taking to cocaine in hope of being lifted out ot the mire, I would say, " don't," lest he sink the deeper.

I have yet to learn of a single instance in which such an effort reached success; but I know many cases where failure followed, or worse, cocaine or coca-morphia addiction.

And the need of caution against free and frequent using obtains in other cases, for there may come a demand for continued taking that will not be denied.

To summarize: Cocaine may be toxic, sometimes deadly, in large doses.

It may give rise to dangerous or even fatal symptoms, in doses usually deemed safe.

The danger, near and remote, is greatest when given under the skin.

It may produce a diseased condition, in which the will is prostrate and the patient powerless—a true toxic neurosis, more marked and less hopeful than that from alcohol or opium.

Such being my belief, I regard Dr. Hammond's statements mistaken, and his conclusions rash and dangerous.

